# The Irish Programme to Eradicate Bovine Viral Diarrhoea Virus—Organization, Challenges, and Progress

**DOI:** 10.3389/fvets.2021.674557

**Published:** 2021-06-01

**Authors:** David Graham, Simon J. More, Padraig O'Sullivan, Elizabeth Lane, Damien Barrett, Jose-Maria Lozano, Hans-Hermann Thulke, Sharon Verner, Maria Guelbenzu

**Affiliations:** ^1^Animal Health Ireland, Carrick on Shannon, Ireland; ^2^Centre for Veterinary Epidemiology and Risk Analysis, UCD School of Veterinary Medicine, University College Dublin, Dublin, Ireland; ^3^Irish Cattle Breeding Federation, Bandon, Ireland; ^4^Animal Health Division, Department of Agriculture, Food and the Marine, Dublin, Ireland; ^5^Surveillance, Animal By-products and TSEs (SAT) Division Department of Agriculture, Food and the Marine, Celbridge, Ireland; ^6^Central Veterinary Research Laboratory, Department of Agriculture, Food and the Marine, Celbridge, Ireland; ^7^Department of Ecological Modelling, Helmholtz Centre for Environmental Research GmbH - UFZ, Leipzig, Germany; ^8^Animal Health and Welfare NI, Unit 49, Dungannon Enterprise Centre, Dungannon, United Kingdom

**Keywords:** Bovine viral diarrhoea virus, eradication, tissue tag, database, retention, model

## Abstract

A mandatory national Irish bovine viral diarrhoea (BVD) eradication programme, coordinated by Animal Health Ireland, commenced in 2013. Key decisions and programme review are undertaken by a cross-industry Implementation Group (BVDIG) supported by a Technical Working Group. Ear notch tissue is collected from all new-born calves using modified official identity tags, supplemented by additional blood sampling, including for confirmatory testing of calves with initial positive results and testing of their dams. Testing is delivered by private laboratories in conjunction with the National Reference Laboratory, with all results reported to a central database. This database manages key elements of the programme, issuing results to herdowners by short message service messaging supplemented by letters; assigning and exchanging animal-level statuses with government databases of the Department of Agriculture, Food and the Marine to enable legislated restrictions on animal movements; assigning negative herd status based on test results; generating regular reports for programme management and evaluation and providing herd-specific dashboards for a range of users. Legislation supporting the programme has been in place throughout but has not thus far mandated the slaughter of persistently infected (PI) calves. A key challenge in the early years, highlighted by modeling, was the retention of PI animals by some herd owners. This has largely been resolved by measures including graduated financial supports to encourage their early removal, herd-level movement restrictions, ongoing programme communications and the input of private veterinary practitioners (PVPs). A framework for funded investigations by PVPs in positive herds was developed to identify plausible sources of infection, to resolve the status of all animals in the herd and to agree up to three measures to prevent re-introduction of the virus. The prevalence of PI calves in 2013 was 0.66%, within 11.3% of herds, reducing in each subsequent year, to 0.03 and 0.55%, respectively, at the end of 2020. Recent regulatory changes within the European Union for the first time make provision for official approval of national eradication programmes, or recognition of BVD freedom, and planning is underway to seek approval and, in due course, recognition of freedom within this framework by 2023.

## Introduction

Bovine viral diarrhoea virus (BVDV) is recognized to be an economically important pathogen that, with few exceptions, is endemic in most countries of the world (1, 2). In some European countries, there has been a focus on control and eradication for more than two decades. In the early 2000's there was extensive debate on the optimum approach to eradication, with this largely characterized by an emphasis on either zoo-sanitary (i.e., identification and removal of persistently infected [PI] animals) or vaccine-led options. A Thematic Network on Control of Bovine Viral Diarrhoea Virus, funded by the European Union (3), brought together researchers from across Europe, who concluded that the key to eradication was not in the debate between these two positions, but rather in the adoption of a systematic approach, comprising identification and removal of PI animals, the application of appropriate biosecurity measures (potentially including vaccination) and ongoing monitoring to ensure that uninfected herds remained free from infection (4, 5).

This systematic approach was pioneered by the Scandinavian countries where programmes based on serological screening of herds through bulk tank milk, first lactation and young stock check tests were used to categorize herds as being likely to be free of infection or, alternatively to contain one or more PI animals. In the latter case, whole herd testing to identify and remove any PI animals was then conducted (6–10). This approach, commonly referred to as the Scandinavian model, has also been adopted on a voluntary or compulsory basis in other countries at either a regional or national level, including Austria (11), Scotland (12, 13), France (14), and the Netherlands (15, 16). More recently, a different systematic approach, based on direct testing of all new-born calves for viral antigen (by antigen-capture ELISA [AC-ELISA]) or RNA (by RT-PCR) has been developed and is commonly known as the Swiss model, since this was the first country in which it was implemented. Its emergence reflected both technological advances, including the development of modified official identity tags capable of collecting ear tissue for testing and the availability of cost-effective virus tests, and epidemiological considerations, including the widespread mixing of cattle from different herds at summer pastures and an associated high seroprevalence within the cattle population (17, 18).

Programmes based on this Swiss approach have subsequently been adopted in other European countries or regions, including Germany (16, 19), Ireland (20), Northern Ireland (21), and Belgium (22).

While many countries in Europe are now in the process of either implementing eradication programmes, or running surveillance programmes to provide ongoing evidence of freedom, these are heterogeneous in nature (16), reflecting differences not only in the context in which they operate (e.g., in prevalence, aims, population size and structure, availability of vaccines, presence of a legislative basis and extent of importation) but also in the testing requirements for enrolment and subsequent surveillance.

Animal Health Ireland (AHI; www.animalhealthireland.ie), a not-for-profit public-private partnership, was established in 2009 (23) to improve the profitability and sustainability of the Irish farming and agri-food sector through improved animal health. Stakeholders include dairy and beef processors, farmer and breed society representative organizations, AI companies, providers of professional, advisory and support services and government and state agencies. An AHI-led BVD Steering Group reviewed options for a possible programme (24), while an economic assessment estimated annual losses to farmers due to BVD of ~€102 million (25). This led to the establishment of a voluntary national BVD eradication programme in 2012, adopting a Swiss-type approach and co-ordinated by AHI [reviewed by (20)]. A compulsory programme commenced in January 2013. This paper provides an overview of the compulsory programme, with a particular focus on its organization, challenges and progress.

## Programme Information

### Governance

Governance structures in the compulsory programme are similar to those that were in place during the voluntary programme. The decision-making body for the programme is a cross-industry BVD Implementation Group (BVDIG), with membership open to representatives of all AHI stakeholder organizations. Currently (early 2021), this comprises the Department of Agriculture, Food and the Marine (DAFM), Glanbia (dairy processor), the Irish Cattle Breeding Federation, the Irish Cattle and Sheep Farmers' Association, the Irish Co-Operative Society, the Irish Creamery Milk Suppliers' Association, the Irish Farmers' Association, the Irish Holstein Friesian Association, the national reference laboratory (NRL), the Pedigree Cattle Breeders Council of Ireland, Teagasc (the national agriculture and food development authority) and Veterinary Ireland. The BVDIG meets regularly, on an approximately monthly basis. A separate Technical Work Group (TWG), with an independent chair (who also sits on the BVDIG), is tasked with providing ongoing scientific advice to the BVDIG and responding to queries generated by it.

### Legislation

The first legislation relating to the programme was introduced in 2012, with new regulations, and amendments to these, introduced subsequently. Each of these is summarized below.

#### The Bovine Viral Diarrhoea Order 2012 (Statutory Instrument [S.I.] 532 of 2012)

The transition from a voluntary to a compulsory programme was enabled by the introduction of this legislation (26), key elements of which comprised:

- Defining tissue tags permitted for use, either as an approved tag (for the purposes of official identification and capable of collecting a tissue sample marked with the identity of the animal sampled) or as a supplementary tag (not an approved tag but otherwise capable of taking a sample marked with the identity of the animal sampled).
- A duty on the farmer to take samples within 20 days of birth from all calves born after 1st January 2013 and to submit these for testing.
- A requirement to submit a repeat sample (collected using a supplementary tag or by blood sampling) where the initial sample was inadequate or missing.
- A requirement to submit a tissue sample from aborted, stillborn or dead calves.
- Provision of the option to re-test after at least 21 days, by supplementary tag or blood sample, any animal that returns an initial positive or inconclusive result, to determine if it is PI or transiently infected (TI).
- A requirement to sample (by supplementary tag or blood) and test animals notified as being suspected of being affected with BVD virus (e.g., dams of calves with positive results).
- A prohibition on the movement of animals born after 1st January 2013 except for disposal as an animal by-product, to slaughter or under permit, unless they has a negative test result.
- A schedule of laboratories designated to provide test results to the programme.

#### The Bovine Viral Diarrhoea Regulations 2014 (S.I. 118 of 2014) (27)

The main changes relative to the BVD Order (2012) were:

- Inclusion of a prohibition on the movement to slaughter of animals that had not been subject to a required test.
- Provision of further detail for the basis of laboratory designation.
- Formalizing the role of the National Reference Laboratory.

#### The Bovine Viral Diarrhoea Regulations (2017) (S.I. 30 of 2017) (28)

The main change relative to the BVD Regulations (2014) was the requirement to conduct re-testing of animals with an initial positive or inconclusive result, or testing of animals notified as being suspected of being affected with BVD, by blood sample only (withdrawing the option to use a supplementary tag).

#### The Bovine Viral Diarrhoea (Amendment) Regulations (2020) (S.I. 182 of 2020)

This amendment (29) provided for the compulsory testing of all bovines born before 1 January 2013, with the exception of those female bovines for which a valid BVD virus test result is recorded for one or more offspring.

### Testing Regime

Details of this, and other programme elements, have been described elsewhere (16). A tissue tag sample must be collected from all calves born since 1st January 2013 and submitted by the farmer for testing to the designated private laboratory of their choice and at their expense.

Laboratories are designated for each test method (AC-ELISA or RT-PCR) and sample matrix (tissue, blood, milk) on the basis of applications submitted to the BVDIG and evaluated primarily by the NRL. These include conditions related to accreditation, turnaround times (currently set at 95% and 99% within 4 working days and 7 working days, respectively, of sample reception) and transfer of results to the programme database in a standard format.

Where a positive or inconclusive result was reported, the animal was considered to be PI until shown otherwise by confirmatory testing (hereafter, these animals, which have been confirmed as PI by re-testing or removed following an initial positive or inconclusive result without re-test, are collectively termed BVD+). Analysis of programme data by the NRL indicated the potential for false negative results in blood samples tested by AC-ELISA due to the interference of maternal antibodies (“diagnostic gap”) in calves <75 days of age and this was therefore set as a lower age limit for testing of this matrix by this method (30, 31). Therefore, blood samples for confirmatory testing were directed to the NRL for testing by methods which addressed this problem (20). Conversely, where tissue samples collected by the farmer using a supplementary tag were submitted for confirmatory testing and returned a negative result, the designated testing laboratory(ies) generating the results were requested to submit the tissue samples to a further laboratory for DNA analysis to confirm that both samples were from the same animal. The confirmatory test was only reported as negative when identity was confirmed. Where this was not possible, including where it was not possible to generate a DNA profile from one or both tissue samples, a blood sample was required to validate the negative tissue sample (20). The dams of animals considered to be PI were themselves deemed to be suspected of being affected with BVD, and as such, they are assigned a DAMPI status (“dam of a PI,” Table 1) and are required to be tested. Between 2013 and 2016 the rules around assigning and managing DAMPI status became increasingly stringent, with a final revision in May 2016 such that all dams with a registered BVD+ calf were assigned a DAMPI status, independent of their previous test history, including a negative tissue tag test as a calf, with a requirement for a subsequent direct negative test to revoke their DAMPI status. Furthermore, any offspring of animals considered to be PI and whose status was not known were also deemed suspect, being assigned an OFFPI status (“offspring of a PI,” Table 1) and are also required to be tested.

**Table 1:** Summary of the 13 possible statuses assigned to each animal in the programme database in relation to its BVD status, and the interpretation and action recommended with each one.

**Status**	**Interpretation**	**Action**
DAMPI	Dam of an animal with a current positive (or inconclusive) result	Test to clarify dam status
Empty	No tissue in submitted sample (unsuitable for testing)	Re-test required. Tissue or blood
Inconclusive	Current inconclusive result on database where initial result was not positive/inconclusive (e.g., initial empty result)	Isolate; option to re-test after 3–4 weeks to confirm PI
INDINEG 1, 2, 3, N	Dam that has produced 1, 2, 3, N negative calves (not PI)	–
INIINC	Initial test result is inconclusive, no re-test result	Isolate; option to re-test after 3–4 weeks to confirm PI. Isolate and remove as soon as possible
INIPOS	Initial test result is positive, no re-test result	Isolate; option to re-test after 3–4 weeks to confirm PI. Consider removal without retest
Invalid	Result not valid	Re-test required. Tissue or blood
NEGATIVE	Tested negative (most recent)	–
NONCOMP35	Animal without any test result 35 days after date of birth Re-test required. Tissue or blood	Test required by legislation
OFFPI	Untested offspring of a dam with a current positive (or inconclusive) result	Isolate and remove as soon as possible
PI	Initial and confirmatory positive (or inconclusive) result	Isolate and remove as soon as possible (<3 weeks of first test)
Positive	Current positive result on database where initial result was not positive/inconclusive (e.g., initial empty result)	Isolate; option to re-test after 3–4 weeks to confirm PI. Consider removal without retest
Unknown	(1) Born before 1st January 2013 and has not been tested and has not calved OR (2) a calf that has been born <35 days ago without any test result	(1) Test to clarify status (result required for Negative herd status if it remains in herd)(2) Test required by legislation

Whilst it is not possible to definitively state based on a single inconclusive or positive test result whether an animal is PI or TI, analysis of the outcomes of confirmatory testing of calves, related to their initial ear notch test values generated by ELISA and PCR test values, indicated that the initial test values were predictive of the outcome of confirmatory testing (32). This analysis was included in the training of private veterinary practitioners (PVPs) for delivery of herd investigations (see section Herd Investigations), enabling them to advise farmers on the merits or otherwise of waiting 3 weeks to conduct a confirmatory test rather than disposing of the animal immediately.

### Financial Supports

Beginning in the voluntary period in 2012, and evolving over the course of the programme, a series of financial supports have been provided by DAFM to farmers, following removal of certain types of BVD+ animals, subject to the terms and conditions for each year. Details of the levels of supports for the years 2012–2015 have been described previously (33). In each of the years 2012–2014, these were paid at a flat rate for removal according to the breed of the calf (dairy or beef) without a specified maximum period of time for removal. Beginning in 2015, the value of these supports was increased but became both graduated and time bound in an effort to promote earlier removal of BVD+ calves, with the maximum level of support available when the BVD+ animal removed within 5 weeks of the initial positive result (€140 and €100 for beef breed calves and dairy breed heifers, respectively), a lower rate paid for removal between 5 and 7 weeks (€90 and €50 for beef breed calves and dairy breed heifers, respectively), and ceasing if removed after 7 weeks.

These supports were maintained at the same level in 2016, but revised in 2017 in terms of their scope, value and time limits, providing €185 or €60 for beef breed animals and €150 or €35 for dairy and dairy cross-breed heifers removed within 3 and 5 weeks, respectively, and introducing a payment of €30 for removal of dairy breed bull calves within 3 weeks.

The levels of supports were unchanged in 2018, but further revised for 2019 and 2020 to provide €220 or €30 for beef breed animals and €160 or €30 for dairy and dairy cross-breed heifers removed within 10 and 21 days, respectively, and €30 for removal of dairy breed bull calves within 14 days.

### Programme Database

The programme database has been developed for AHI by the Irish Cattle Breeding Federation (ICBF^1^). The basic unit within the database is the individual animal, identified by its official identity number assigned by ear tagging and recorded in the Animal Identification and Movement System (AIMS) database of DAFM. Each animal in turn is located within a specific herd, which again has a unique identifier assigned by DAFM, with all movements of animals also recorded on AIMS, which on this basis maintains a current listing of all animals in each herd. These animal- and herd-level data (including dam details) are also shared with ICBF on a daily basis to enable programme management.

#### Test Results

These are received on a daily basis from designated laboratories and associated on the database with the relevant animal and herd. Based on these results, the database assigns one of 13 possible, mutually exclusive statuses, to each individual animal (Table 1), taking into account both its own test results and those of its offspring and dam (e.g., assigning an indirect negative status [INDINEG] to a dam on the basis of a direct negative result for a calf). For each animal, all test-associated information is retrievable, including the date of test, the sample type, the testing laboratory, the test method, the test value generated and the interpreted result. Test results and animal statuses are also passed back to AIMS, with these enabling the movement controls laid down in legislation. The database also manages communication of results to herd owners, with these typically being issued as short message service (SMS) messages to their mobile telephones on the day of receipt. In addition, the database automatically generates a series of result-driven letters e.g., following an initial positive or inconclusive result, providing more detailed information and guidance, along with a pre-populated submission form in cases where further testing may be appropriate.

#### Dashboards

For each herd-owner, a dashboard has been developed, providing access to all results in the database for the farm in question, a real-time summary of the entire herd by status, and an archive of all letters issued over the course of the programme. These dashboards are also available to AHI and PVPs for programme management.

The dashboards also provide a series of additional options, with these being particularly useful for herd investigations following positive results. These include:

##### Purchase History

For either a selected or all years of the programme, and for each introduced animal, this option lists the date of birth, date of introduction, current age, date of departure from the current herd (where relevant), birth herd, most recent test date and status and whether in calf at purchase (based on first recorded calving date after introduction) and, where relevant, the test status of this calf.

##### Investigate Function

Based on a window of susceptibility (WOS) of 30–120 days of gestation for establishing persistent infection *in utero*, and on the recorded date of birth, this option presents the following information for each animal with an initial positive or inconclusive (INIPOSINC) result (for either a selected or all years of the programme): data of birth, date of 30th day of gestation, date of 120th day of gestation, date and results of first and any subsequent tests and, where relevant, the date of removal from the herd. For the dam of each INIPOSINC animal, the following are listed: date of birth, whether homebred or not, date of entry to the herd, entry date, the interval from entry to calving, and test history. In addition, this screen gives access to a family tree function showing the ancestors or descendants of a given animal by sex, date of birth, date of death, and status.

##### Contiguous Herds

For herds with BVD+ animals, this option provides details of the total number of contiguous herds (i.e., those with which the case herd shared a common boundary), the number of these that have had animals with INIPOSINC results, and the dates of birth and death of each of these animals.

#### Programme Reports

In addition to the functionality already described, the database also provides access to a series of additional outputs that are used for programme management and monitoring and are generated as standard outputs that are available to download, are issued as regular reports, or both. These include:

##### Daily List of INIPOSINC Results

Telephone calls to herdowners following their first positive or inconclusive result in a given year are made by the BVD Helpdesk, which is staffed by DAFM personnel. These calls are complementary to the other programme communications already described (SMS, letter) and are intended to ensure that the herdowner is aware of the result, its implications, available financial supports, and the requirements for a herd investigation (see below), including recording the details of the PVP nominated to conduct this.

##### Weekly Updates at Animal and Herd Level

These updates provide the basis for a weekly programme summary^2^ including summary annual figures and key statistics for the current year alongside those for the equivalent week during the preceding year. In addition, monthly maps showing the distribution and number of BVD+ births, the number of identified BVD+ animals alive and the number retained (see section Herd Restrictions for definitions of retention) are published on a monthly basis.

##### Weekly List of Animals With Apparent False Negative (AFN) Results

In the context of the programme, an AFN result occurs when an animal returns a positive or inconclusive result having recorded a previous negative result. The database identifies each such occurrence and generates a cumulative report, including details of any further testing, for further analysis. The profile of the results or the statuses recorded by the database for each animal with an AFN result is determined and updated as further results become available, using the following signifiers for each result/status: N, negative; P, positive, I, inconclusive; D, DAMPI. For example, a profile of N-P-P signifies an animal with an initial negative result and two subsequent positive tests.

##### Laboratory Performance

For each designated laboratory and test method, the database provides the percentage of results that have been returned within 4 and 7 days, and highlights where these exceed those specified in the designation criteria.

##### Assignment and Management of Negative Herd Status (NHS)

While the programme focuses on the status of individual animals, it also assigns NHS to herds which satisfy the following 3 conditions:

- completion of a minimum of 3 years of tissue tag testing on calves born into the herd,
- existence of a negative BVD status for every animal currently in the herd (on the basis of either “direct” or “indirect” results),
- absence of any BVD+ animal(s) from the herd in the 12 months preceding the acquisition of NHS.

Maintaining NHS requires herds to continue to satisfy the second and third of these requirements. NHS is withdrawn after a defined period following the purchase of one or more animals with an UNKNOWN status (unless tested for BVD after purchase, with a negative result); failure to conduct any testing required following notification of suspicion of infection with BVDV (e.g., introduced animals assigned a DAMPI or OFFPI status) or animals with empty or invalid results from initial testing, or the detection of a PI animal.

Herd owners are notified by SMS when NHS is first assigned, and subsequently the database issues a series of SMS alerts and reminder letters to herdowners prior to withdrawal of NHS due to failure to conduct necessary testing.

While acquisition of NHS is a milestone for each herd in terms of disease control, it also affords access to testing at reduced cost through a number of designated laboratories, reflecting the greatly reduced likelihood for a positive result when testing pooled samples by RT-PCR with the consequent requirement for further testing of individual samples.

For analysis purposes, herds without NHS are considered as either NHS-U (satisfy the first and last requirements but contain one or more animals whose status is not known) or NHS-P (currently contain one or more BVD+ animals, or have done so in the preceding 12 months, with or without additional animals whose status is not known).

### Herd Investigations

Beginning in 2016, herds with BVD+ animals were required to undergo an investigation within 3 months of the initial positive result. These are coordinated by AHI and delivered by a cohort of more than 540 trained private veterinary practitioners (PVPs). The investigations are co-funded by DAFM and the European Commission through the Rural Development Programme (2014–2020) as one component of a Targeted Advisory Services on Animal Health (TASAH). These investigations have the 3-fold purpose of seeking to identify one or more plausible explanations for the BVD+ birth, ensuring that all BVD+ animals in the herd have been identified and removed (including testing of any animals whose status is not known) and to review herd biosecurity. Based on the findings, up to three measures are agreed to be implemented by the herd owner to reduce the risk of re-introduction of infection.

Beginning in 2019, these investigations were enhanced by requiring targeted testing of not only those animals that did not have a known BVD status, but also those that had only one negative result (direct or indirect) recorded on the programme database and that were present in the herd during the WOS of the dam(s) of the BVD+ animal(s).

Further details of the structure and findings of these investigations will be reported elsewhere (Guelbenzu-Gonzalo and Graham, Front Vet Sci submitted).

### Retention of BVD+ Calves and Development of a National Model

A review of the voluntary phase of the programme indicated that while the majority of herd owners removed BVD+ calves, 26.5% of those born in the study period (1st January to 15th July, 2012) were still alive at its end (20), with a disproportionate number of these in beef herds. A subsequent study highlighted the non-removal of these calves as one factor significantly associated with retaining herds having further BVD+ births the following year (34). The importance of prompt removal of BVD+ animals to the progress of the compulsory programme was therefore a key feature of programme communications. From 2013 to 2016, a BVD+ animal was deemed to retained if it was still alive more than 49 days after the date of its initial positive or inconclusive test. During this period the proportion of BVD+ animals removed within 7 weeks increased each year from 43.7% in 2013 to 70.3% in 2015 (33), but still fell well short of the 100% target.

To further explore the impact of retention, and assist decision making by the BVDIG, an expert system model (FarmNet-BVD) was developed (35) and used to model the impact of scenarios with various times to removal, implemented from 2017 onwards, on projected times to eradication. Key findings from this work were that eradication was not achievable within a realistic time window if retention continued at the levels seen in 2015, in contrast to the outcomes under various scenarios whereby all BVD+ were removed within 7 weeks or less.

### Vaccination

At the beginning of the eradication programme, two inactivated vaccines Bovidec (Novartis Animal Health) and Bovilis BVD (MSD Animal Health) were licensed for use in Ireland (although the former is no longer marketed). A live vaccine (Bovela, Boehringer Ingelheim) was subsequently licensed in 2016. The BVD TWG has issued guidance on the role of vaccination in the programme, but decisions on whether to begin, maintain or cease vaccination against BVD rest with the herd owner and their PVP. While vaccination history is explored in the context of herd investigations, there is not currently a mechanism to routinely record vaccination at either herd or animal level on the database. However, details of total annual vaccine sales were obtained through a market research company^3^ and analyzed for changes over time.

### Additional Measures to Prevent Spread

#### Herd Restrictions

Beginning in 2016, DAFM began imposing restrictions on both inward and outward movements (except to slaughter) on herds retaining BVD+ animals (not removed with 49 days of the initial result), with these being lifted immediately on removal of the retained animal(s). The initial focus was on herds with retention periods exceeding 12 months, but from 2017 onwards, these restrictions were automated and applied as soon as the herd was determined by the database to be a retention herd. In 2017, aligned with changes to the financial supports, animals were considered to be retained if not removed within 35 days, with a further reduction to 21 days from 2019 onward.

#### Neighbor Notifications

Associated with the imposition of movement restrictions, herds contiguous to the retaining herd were notified that a BVD+ animal was being retained in a neighboring herd and advised to ensure that biosecurity measures were in place to minimize the risk of accidental introduction of infection.

## Progress Toward Eradication

### Cattle Population, Testing Profile, and Outcomes

At the end of 2020, the programme database contained information on 82,211 herds containing a total of 5,525,732 cattle. These were categorized as beef (59,501), dairy (17,708) or dual (dairy and beef enterprise; 5,002). The mean and median number of cattle in each of these three herd types was 44 and 29 (beef), 154 and 124 (dairy) and 99 and 64 (dual), respectively.

**Figure 1:**
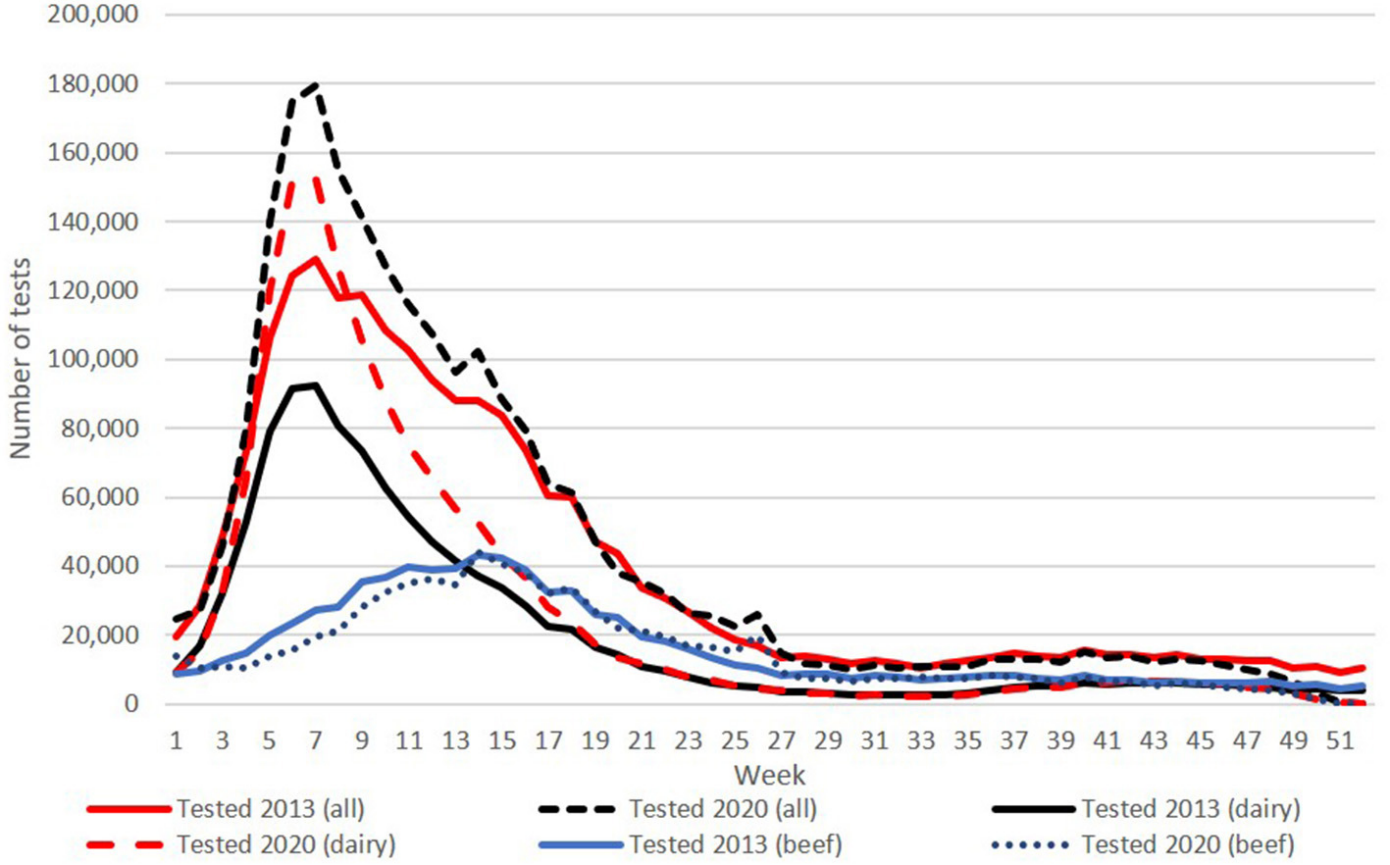
Number of tests conducted per week in 2013 (solid lines) and 2020 (broken lines) for all calves and, separately, for calves born in either dairy herds or beef herds.

A total of 2,381,730 calves were registered with a date of birth in 2020 (accessed 28.01.21), with a BVD test result recorded for 99.5% of these (2,366,532). Consistent with the predominantly spring-calving profile of Irish cattle herds, there was marked seasonal variability in the number of samples tested each week, with an overall peak of 179,471 in week 7 (Figure 1), coinciding with the peak week of testing for calves born in dairy herds (152,138). This spring-calving profile was also evident in data from beef herds, although the curve was flatter and the peak (44,252) occurred later, in week 14. Except for week 40 (15,152 total tests in 2020), weekly numbers were below 15,000 from week 27 onward. This pattern was consistent throughout the programme, although absolute numbers were higher in 2020 (with a total of 2,095,892 calves tested in 2013), reflecting a 33% increase and an 8% decrease in tests on calves born in dairy herds and beef herds, respectively.

Despite this marked seasonality in test volumes, laboratory performance was consistently within agreed targets, with (for 2020) a median interval from receipt to reporting of 1.1 days, and 99.5 and 99.8% of results reported within 4 and 7 working days, respectively.

**Figure 2:**
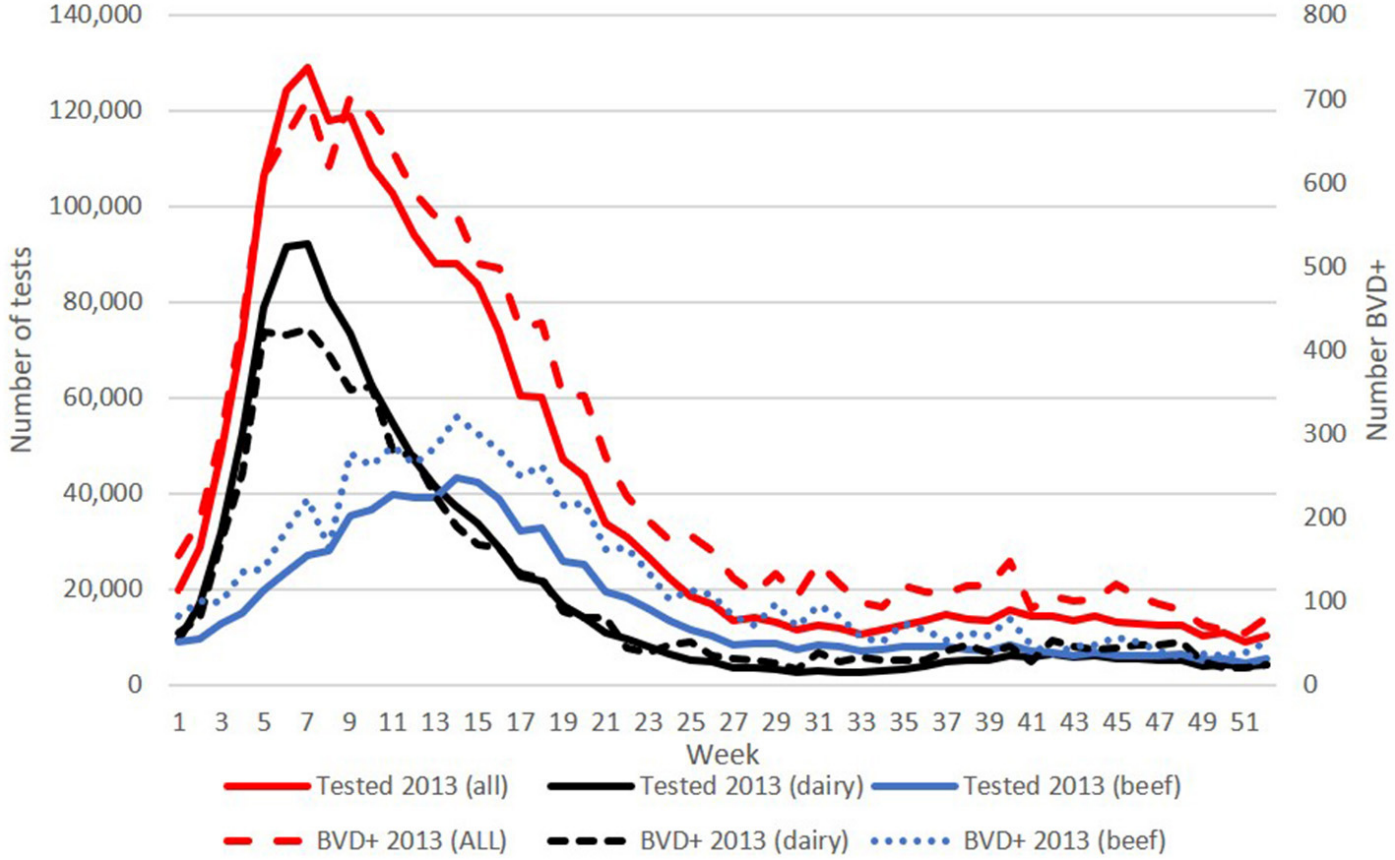
Weekly number of calves tested (left y-axis) overall, and from dairy and beef herds in 2013 (solid lines), and the corresponding number of BVD+ calves (right y-axis) detected each week (broken lines). BVD+ calves are those with an initial positive or inconclusive result without a negative retest result.

The numbers of BVD+ calves detected on a weekly basis in each year of the programme reflected the calving profile, with highest numbers born each spring. For example, in 2013 over 700 BVD+ calves were born in weeks 6 and 8, declining to around 100 from week 29 onwards (Figure 2).

**Figure 3:**
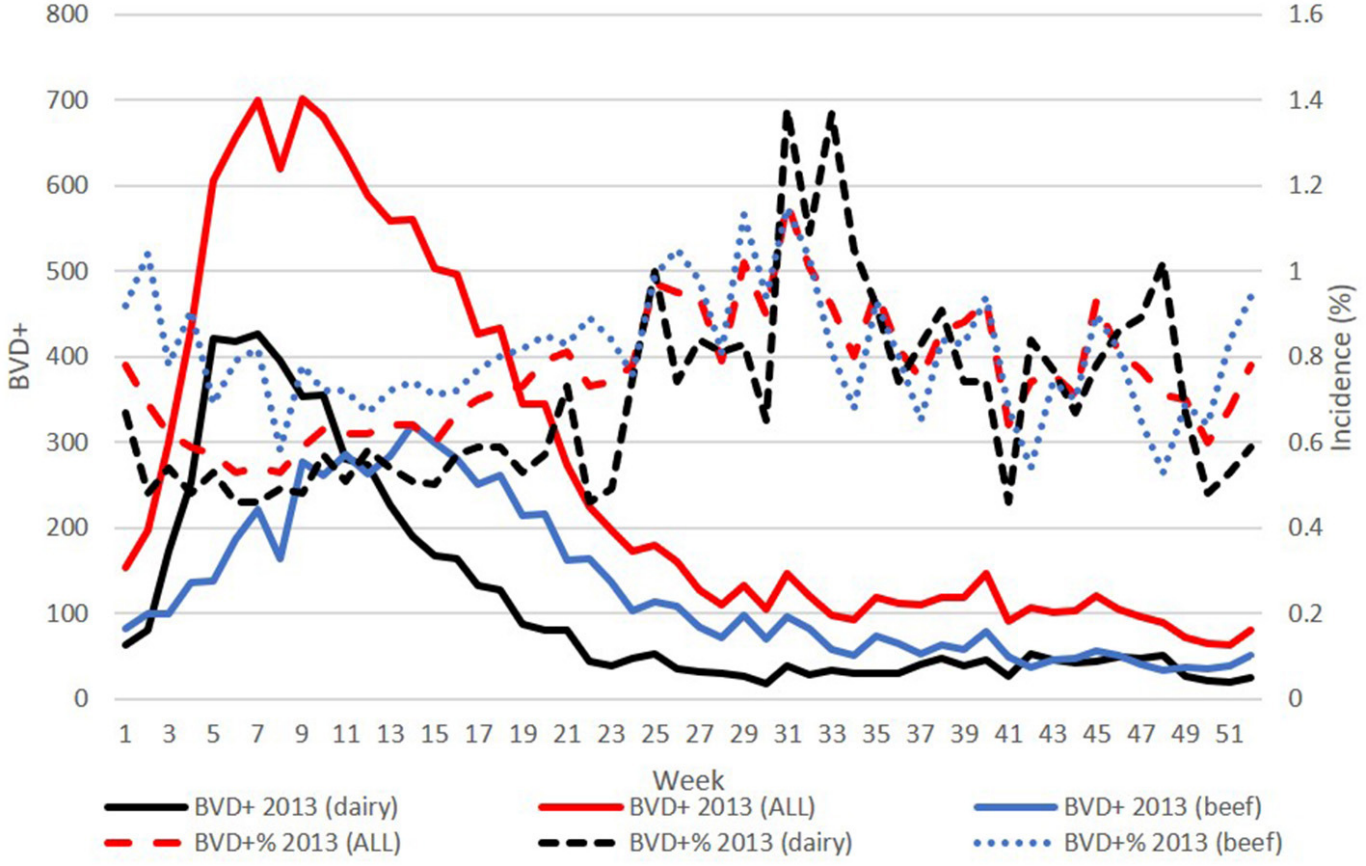
Plots of weekly number (left y-axis) overall, and from dairy and beef herds in 2013 (solid lines), and the corresponding weekly incidence of BVD+ births (right y-axis) detected each week (broken lines). BVD+ calves are those with an initial positive or inconclusive result without a negative retest result.

However, when the incidence of BVD+ calves detected each week was assessed, it was found that this was lowest in the spring, while the highest incidence occurred, independent of herd type, later in the year around weeks 30–35. This pattern was most pronounced in the early years of the programme (illustrated for 2013 in Figure 3) but was evident each year in both dairy and beef herds.

**Figure 4:**
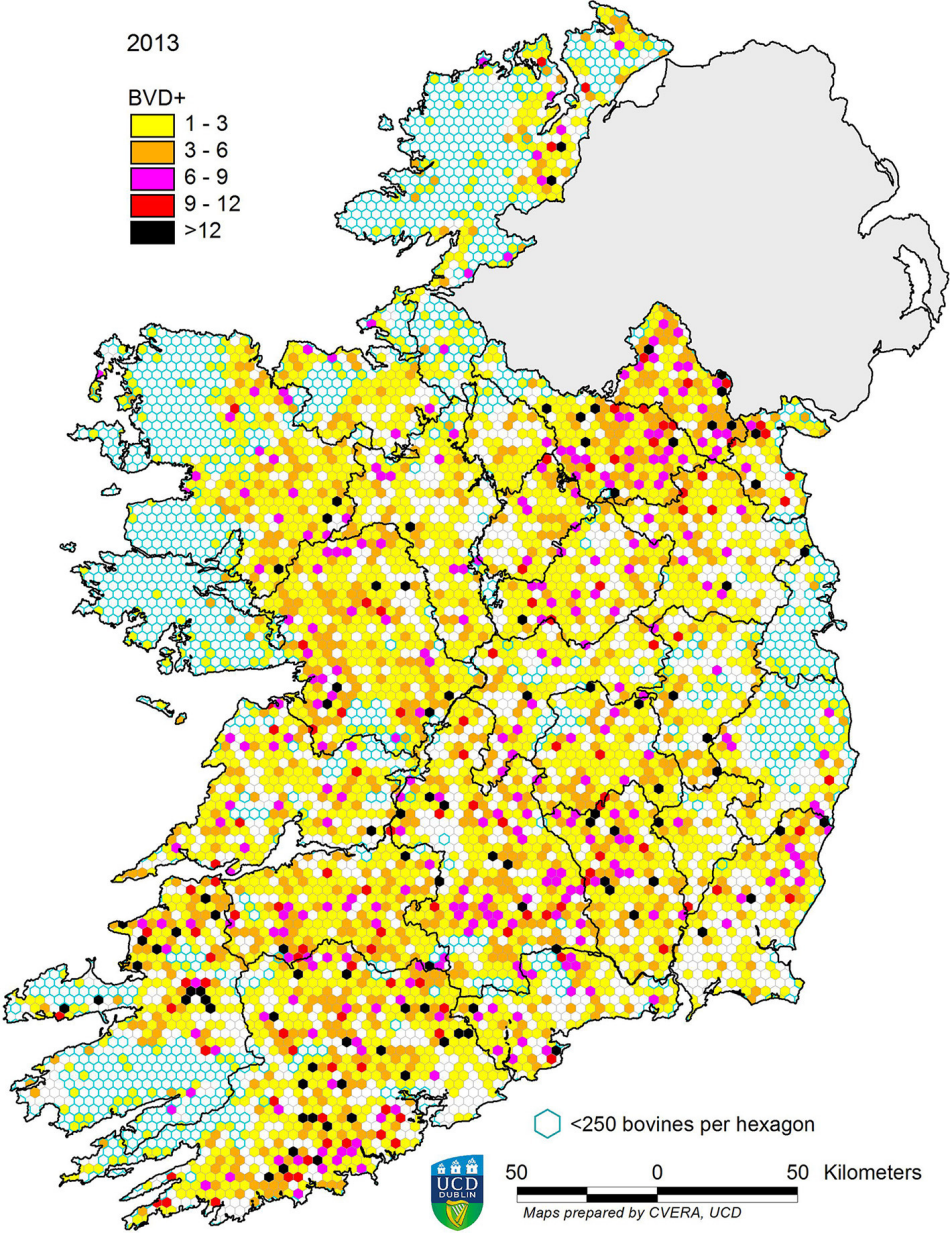
Distribution of BVD+ calves born in 2013. Each hexagon ~10 km^2^.

**Figure 5:**
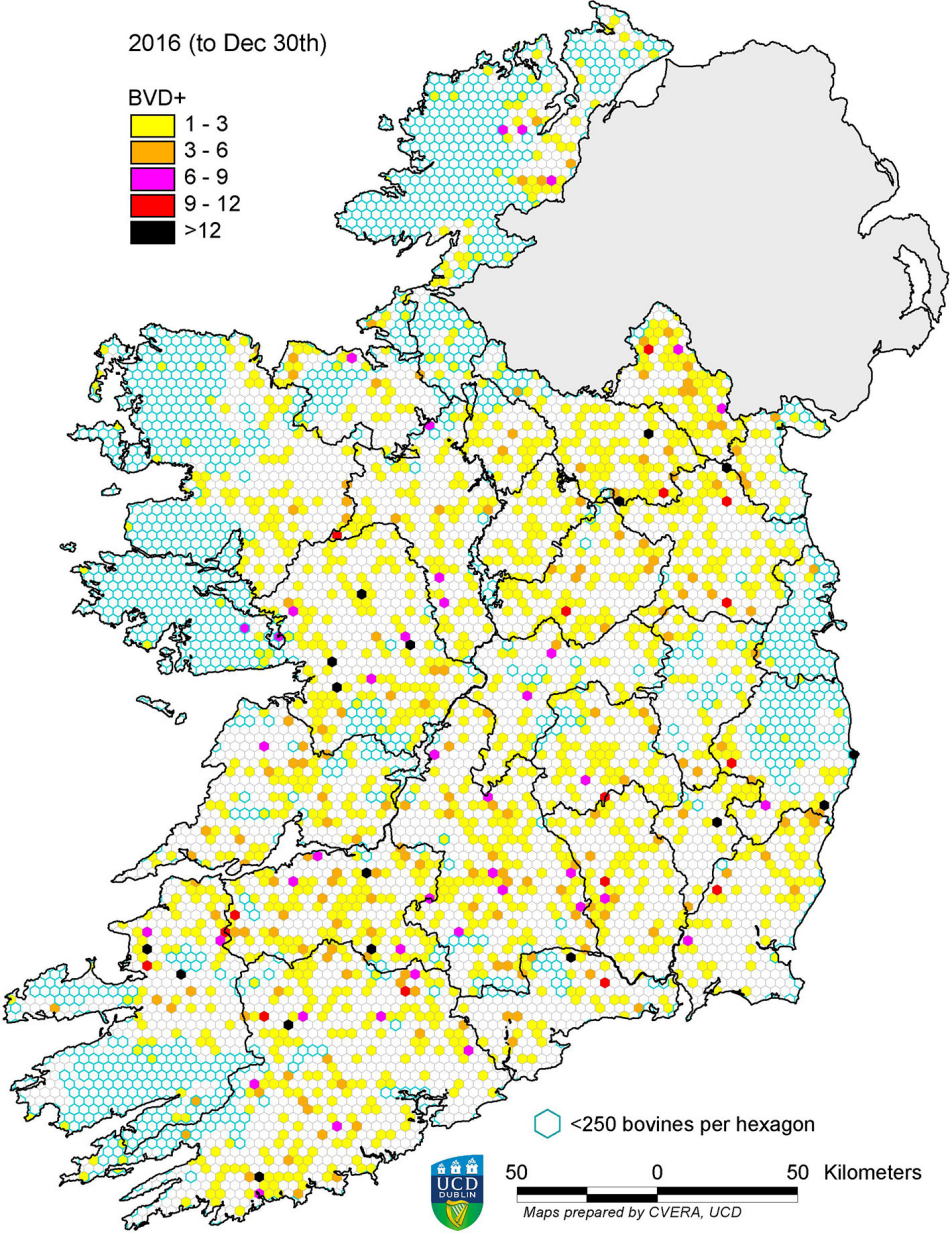
Distribution of BVD+ calves born in 2016. Each hexagon ~10 km^2^.

**Figure 6:**
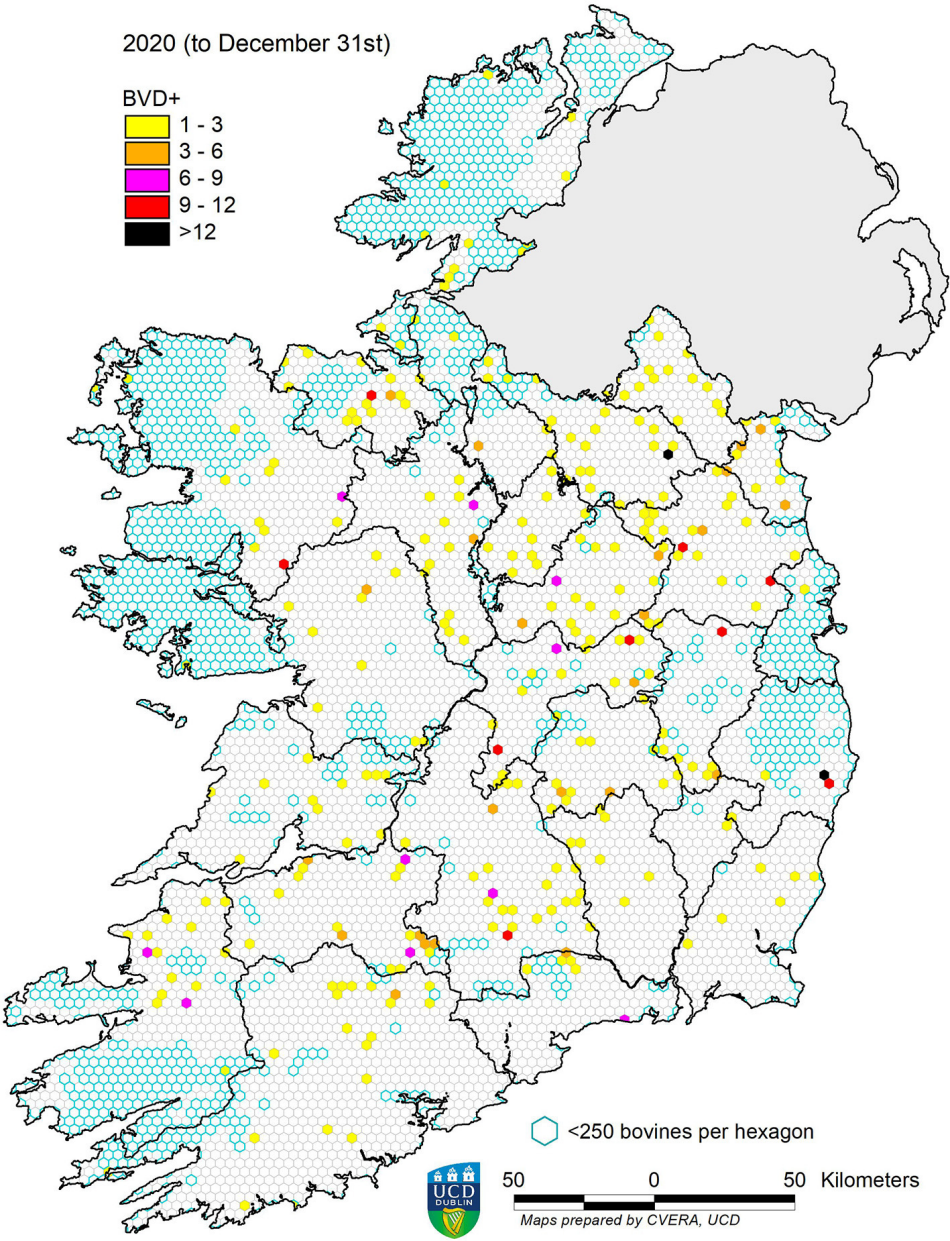
Distribution of BVD+ calves born in 2020. Each hexagon ~10 km^2^.

In 2013, 13,877 BVD+ calves were detected, representing a prevalence of 0.66%. This figure decreased in each subsequent year, to 0.03% in 2020 (Table 2). The spatial changes associated with this reduction in prevalence are illustrated for the years 2013, 2016, and 2020 in Figures 4–6, respectively. A similar pattern of reduction was observed at herd level, with positive or inconclusive results recorded in 9,484 herds (11.27%) in 2013, declining to 0.55% in 2020. The recorded animal-level prevalence was higher in beef herds than in dairy herds each year (beginning at 0.78 and 0.55%, respectively, in 2013), while the herd-level prevalence was higher in dairy herds than in beef herds each year (beginning at 20.34 and 8.75%, respectively, in 2013) (Table 3).

**Table 2:** Summary of full-year results for calves born in each year of the programme.

	**2013**	**2014**	**2015**	**2016**	**2017**	**2018**	**2019**	**2020**
Tested	2,095,892	2,131,970	2,264,881	2,325,281	2,347,597	2,346,947	2,343,531	2,366,532
% Negative	98.03%	98.54%	98.85%	99.20%	98.85%	98.58%	98.96%	99.10%
% (Number) Positive*^a^*	0.77% (16,193)	0.50% (10,758)	0.36% (8,247)	0.20% (4,540)	0.12% (2,843)	0.07% (1,531)	0.05% (1,111)	0.03% (790)
% (Number) Inconclusive*^a^*	0.03% (661)	0.01% (119)	0.01% (207)	0.00% (59)	0.01% (118)	0.00% (47)	0.00% (15)	0.00% (15)
% (Number) Empty	1.13% (23,750)	0.92% (19,676)	0.73% (16,637)	0.59% (13,721)	1.01% (23,715)	1.33% (31,132)	0.94% (22,065)	0.81% (19,013)
% (Number) BVD+^b^	0.66% (13,877)	0.46% (9,733)	0.33% (7,427)	0.16% (3,808)	0.10% (2,397)	0.06% (1,325)	0.04% (987)	0.03% (707)
% (Number) of positive herds^c^	11.27% (9,484)	7.63% (6,191)	5.9% (4,770)	3.25% (2,549)	2.03% (1,613)	1.13% (865)	0.78% (571)	0.55% (392)
Median days to removal of BVD+	53	42	32	29	13	12	7	6
% BVD+ retained^d^	52%	42%	27%	20%	15%	17%	24%	18%

a *Based on initial tag test result, prior to any confirmatory testing*.

b *Calves with an initial positive or inconclusive result without a negative retest result*.

c *Based on one or more initial positive or inconclusive tissue tag results for calves born each year in breeding herds*.

d *Retained if not removed within 49 days (2013–2016), 35 days (2017, 2018), and 21 days (2019, 2020), respectively*.

**Table 3:** Animal-level prevalence (%) of BVD+^a^ calves detected each year overall, and by herd type and the prevalence (%) of herds with one or more calves with positive or inconclusive results each year (overall and by herd type).

	**Calf Prevalence**				**Breeding Herd Prevalence**			
**Year**	**Total**	**Beef**	**Dairy**	**Dual**	**Total**	**Beef**	**Dairy**	**Dual**
2013	0.66	0.78	0.55	0.80	11.30	8.75	20.30	14.05
2014	0.46	0.54	0.37	0.60	7.60	5.94	13.22	11.04
2015	0.33	0.39	0.26	0.52	5.94	4.44	10.40	9.29
2016	0.16	0.21	0.12	0.23	3.26	2.39	5.72	5.14
2017	0.10	0.13	0.08	0.17	2.03	1.36	3.90	3.54
2018	0.06	0.07	0.04	0.09	1.13	0.76	2.19	1.93
2019	0.04	0.05	0.03	0.08	0.78	0.52	1.43	1.66
2020	0.03	0.04	0.02	0.06	0.55	0.38	0.96	1.13

a *Calves with an initial positive or inconclusive result without a negative retest result*.

**Figure 7:**
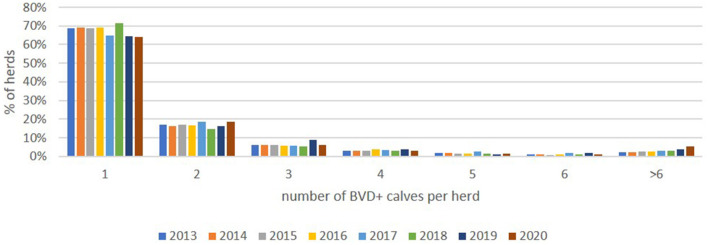
Distribution of the proportion (%) of positive herds each year with from 1 to 6 and >6 BVD+ calves (these being calves with an initial positive or inconclusive result without a negative retest result).

Most commonly, herds with BVD+ calves had only one such detected (64.2%, 2020), with the majority of herds (93.5%, 2020) having 5 or fewer, with this pattern being relatively consistent from 2013 onwards (Figure 7).

While the majority of BVD+ animals detected were calves, smaller numbers of older animals were also detected, including several born prior to 2013 whose ages ranged from 7 to 15 years at the time of detection and which were primarily located in beef herds. The oldest detected was a 15 year old female born in 2001 identified in 2016 when tested as a DAMPI subsequent to its calf testing positive.

### Vaccine Sales

Data on vaccine sales in 12 month periods from July 2016 to June 2020 were available. During 2016/17, a total of 895,450 doses were sold, of which 96.0% were inactivated. Total doses sold decreased in each subsequent period by 7.4, 22.0, and 12%, respectively (32.2% overall; 607,415 doses), with the proportion of inactivated doses sold decreasing slightly from 96.0% in 2016 to 92.4% in 2020.

### Apparent False Negative Results

At the end of 2020, a total of 260 animals born between 1st January 2013 and 31st December 2020 had been identified as AFNs, of which only the 3 most recently detected (15th October 2020 onwards) remained alive. Their years of birth and detection are summarized in Table 4, with the highest number (57) born in 2013 and detected (48) in 2017.

**Table 4:** Number of animals assigned an apparent false negative (AFN) status with the programme, according to years of birth and detection.

	**Year of detection**								
**Year of birth**	**2013**	**2014**	**2015**	**2016**	**2017**	**2018**	**2019**	**2020**	**Total**
2011	4		1						5
2012	12	6	2	1	2				23
2013	14	8	16	15	4				57
2014		6	9	8	11	4	1	1	40
2015			7	6	21	7	1	1	43
2016				2	7	4	1		14
2017					3	3	10	3	19
2018						21	9	10	40
2019							4	12	16
2020								3	3
**Total**	**30**	**20**	**35**	**32**	**48**	**39**	**26**	**30**	**260**

Most commonly (*n* = 155) these had a test profile of N-P, indicating a single positive result recorded following an initial negative result, with smaller numbers having an N-P-P profile (*n* = 23) or a N-P-P-P profile (*n* = 4). Fifty one animals had an N-D-P profile recorded, indicating an initial negative result followed by a DAMPI status and a single positive result on a subsequent test, while 9 had a N-D-P-P profile. All 60 of these animals were detected from 2016 onwards, of which 15 were identified in 2016, 27 in 2017, 13 in 2018, 2 in 2019 and 3 in 2020. The profiles of the remaining 18 AFN animals comprised a range of other test combinations, with a most recent positive or inconclusive result.

Overall, during these 7 years a total of 472,544 animals had an initial positive or inconclusive result; when added to these figures, these 260 AFN animals would represent 0.55% of the overall total.

### Time to Removal and Retention

The median interval between initial positive result and removal for calves born in 2013 was 53 days, with 52% of BVD+ calves born in 2013 being retained (Table 2). The median days to removal decreased in consecutive years, to 6 days in 2020. The proportion of BVD+ calves born each year that were retained followed also declined over this period, though less regularly (Table 2). The lowest proportion of 15% was a achieved in 2017, with values of 17, 24, and 18% in 2018–2020, respectively.

### Confirmatory Testing

The proportion of calves with an initial positive result that were subject to confirmatory testing decreased over the course of the programme (from 74% in 2013 to 35% in 2020), as did the proportion of these that were confirmed as positive (Table 5), decreasing from a maximum of 86% in 2014 to 61–64% from 2016 onward.

**Table 5:** Total numbers (%) of calves born each year that were subject to confirmatory testing, and the number (%) confirmed as positive.

**Year of birth**	**Initial positive**	**Retested**	**% retested**	**Retest positive**	**% Retest positive**
2013	17,276	12,868	74%	9,995	78%
2014	11,109	7,871	71%	6,769	86%
2015	8,593	5,759	67%	4,796	83%
2016	4,680	3,115	67%	2,346	63%
2017	3,014	1,439	48%	873	61%
2018	1,613	772	48%	485	63%
2019	1,152	400	35%	252	63%
2020	798	280	35%	180	64%

### Negative Herd Status (NHS)

By the end of 2020, the majority of animals in the national breeding herd (96.8%) had a direct negative result recorded on the database, while a further 2.8% had an INDINEG status. When considered at the herd level, and taking into account the date of removal of any previous BVD+ animals, 95.3% of these herds had been assigned NHS, with a further 4.2% assigned NHS-U due to the presence of one or more animals aged more than 35 days whose status was not known (Table 6). Collectively, across herds with either NHS-U or NHS-P, there were 10,109 such animals (a mean of 2.6 per herd), the majority of which (75%) were born in 2020 and distributed approximately equally between male and female and beef (50%) vs. dairy/dual (40%/10%) herds. Seventeen percentage of these animals were born prior to 2013 and were predominantly male (75%) and located in beef (50%) or dual (24%) herds.

**Table 6:** The number (%) of breeding herds assigned negative herd status (NHS), NHS-U^a^ and NHS-P^b^ overall and by herd type at the end of 2020.

	**Beef (%)**	**Dairy (%)**	**Dual (%)**	**Total (%)**
NHS	57,179 (96.1)	16,532 (93.4)	4,658 (93.1)	78,369 (95.3)
NHS-U	2,132 (3.6)	1,045 (5.9)	307 (6.1)	3,484 (4.2)
NHS-P	190 (0.3)	131 (0.7)	37 (0.7)	358 (0.4)
Total	59,501 (100)	17,708 (100)	5,002 (100)	82,211 (100)

a *A herd without negative herd status, because of the presence of one or more animals without negative BVD status, on the basis of either direct or indirect results*.

b *A herd without negative herd status, because of the presence of one or more BVD+ animals, either currently or during the preceding 12 months, with or without additional animals whose status is not known*.

## Discussion

The launch of a national BVD eradication programme in Ireland was a significant step for the Irish cattle industry, being the first time that a diverse range of stakeholders, encompassing both private and public sectors, had come together to take a leadership role in addressing a prioritized endemic, non-regulated disease. This approach is recognized as a new departure in biosecurity governance, with a number of associated challenges (36). The initiation and successful implementation of the programme also required a series of logistical and operational challenges to be addressed, particularly those relating to testing and data handling.

Prior to the commencement of a national programme, the laboratory capacity available on the island of Ireland to deliver BVD virus testing to an accredited standard was limited and inadequate relative to programme requirements. A key factor in the success of the programme has been the response by a series of private sector laboratories, guided by the National Reference Laboratory, to this challenge, developing the capacity, in a short period from 2011, to test in excess of 2.3 million tissue tag samples each year to an ISO 17025-accredited standard. This response is particularly notable given the fluctuation seen in sample numbers at the peak of calving in February each year relative to the second half of each year, with this disparity in throughput increasing from 2013 to 2020 as both absolute calf numbers and the magnitude of the spring peak increased over this period (Figure 1).

The challenge of successfully managing these large volumes of data was also critical element of the programme. This was delivered by the programme database developed by ICBF. The reporting of results by all laboratories, in a standard format, to this database was key to efficient data handling within the programme. This enabled prompt reporting of results to herd owners by SMS, within hours of upload the testing laboratory; facilitated the delivery of any further information to be issued following a non-negative result; assignment of a range of statuses at both animal (Table 1) and herd levels; and the control of the movement of animals that did not have negative test results.

As reported previously (20), the success of these programme elements during the voluntary phase of the programme in 2012, along with the degree of participation and support from herd owners (37), were critical to the decision by the BVDIG to request DAFM to introduce legislation to progress to a compulsory programme for 2013. As described, the legislation is straightforward, reflecting the structure of the programme itself.

However, while compliance with the legislative requirements in terms of testing was consistently high, a key challenge in the early years of the programme was the retention of BVD+ animals. Despite epidemiological studies showing that these animals had reduced likelihood of survival and performance (34) and the increased likelihood of additional BVD+ calves being born in these herds in the following breeding season (38), a proportion of farmers chose to retain these animals, attempting to rear them to slaughter weight. Thus, while a significant reduction in the prevalence of BVD+ births was achieved between 2013 and 2014 (Table 3), almost as many 2013- as 2014-born BVD+ animals were alive each month in the spring of 2014, with this most evident in the cohorts born in beef herds (33), with 52% and 42% of BVD+ calves born in these years retained for 49 days or more (Table 2).

The higher incidence of BVD+ births occurring to dams that calve in the second half of the year, as distinct from the peak numbers of BVD+ births each spring, is consistent with these dams being exposed to a higher infection pressure during their WOS, which overlaps with the spring period when the majority of BVD+ calves are born, and retained, each year.

Despite continued retention of BVD+ calves born in 2015 and 2016 by some herd owners, albeit at reducing frequency (Table 2), further reductions in prevalence were achieved, but at slower rates than were acceptable to the BVDIG. The development of the individual-based model^4^ and communication of the results (35), was critical in driving change within the programme to address this issue from 2016 onwards. When first conceived and communicated, it was anticipated that a significant reduction in prevalence would be achieved by the programme within 3 years, offering herds the option to progress from routine tissue tagging to alternative, lower cost, surveillance strategies thereafter. While clearly not achieved, the model outputs confirmed to the BVDIG that, with full compliance in terms of prompt identification and removal of BVD+ calves, this would have been achievable. Further, it demonstrated that if the level of retention seen in 2015 continued in subsequent years, eradication would not be achieved within an acceptable time scale. This contrasted with other scenarios which incorporated prompt removal.

These modeling outputs were the catalyst for the introduction of a series of measures which, alongside other changes introduced since 2016, have largely resolved this issue, such that at the end of 2020 only 5 herds contained BVD+ calves more than 3 weeks after their date of detection. The introduced changes to financial supports and associated measures, along with continued programme communications have played a central role in this change. On one hand, the levels of financial supports have increased, being targeted toward the beef sector where retention was particularly problematic. On the other, the period for which these were available was reduced to 5 weeks in 2017 and again to the current maximum of 3 weeks and front-loaded with a lower rate after 10 days to encourage removal without retesting. This was supported by the analysis of tissue tag test values, demonstrating the correlation between these and the outcome of confirmatory testing (32), enabling PVPs to advise on the merits or otherwise of immediate removal rather than retesting.

In addition, the change in legislation to require all confirmatory tested to be carried out on blood samples ensured that the herd's PVP was involved in the decision-making process. This was further enhanced by the introduction of the TASAH in 2016, which provided the funding for over 540 PVPs, trained by AHI, to deliver in-depth epidemiological investigations of each herd. This ensured that best practice advice was available to each herd, including the importance of prompt removal.

In association with changes to financial supports, herd restrictions were introduced for herds retaining BVD+ animals, with the interval permitted between detection and removal before these were implemented decreasing from 5 to 3 weeks in 2019. From an epidemiological perspective, these restrictions reduced the risk of further onward dissemination of infection from these herds through trade, while also preventing the introduction to the herd of potentially naïve, susceptible animals, either in calf or intended for breeding.

Herds contiguous to BVD+ herds were found to be at increased risk of themselves having BVD+ calves the following season (39), and this informed the decision to implement biosecurity notifications to these herds when the index herd became a retaining herd. However, while the findings of TASAH investigations regularly identified transboundary transmission as a plausible transmission pathway (Guelbenzu-Gonzalo and Graham, submitted), it is recognized that other pathways may be involved and that neighborhood risk operates at a larger scale, with one study reporting an increased risk related to the presence of BVD+ animals within a 10 km radius (40).

Collectively, the impact of these changes are reflected in the reduction in the median time to removal of BVD+ calves and the proportion of these which were considered retained each year (Table 2). Step changes in the time to removal aligned to changes in the programme measures (primarily in financial supports) are evident between 2016 (29 days) and 2017 (13 days), and again between 2018 (12 days) and 2019 (7 days). Marked changes in the proportion of BVD+ calves retained each year is also evident, from 52% in 2013 to 18% in 2020. While the figures in Table 2 indicate that the proportion retained has been relatively stable since 2016 onwards (in the range 15–24%), it is important to note the changes in the number of days after which a calf was considered to be infected across this period. For example, the 15% retained in 2017 are counted after 35 days, whereas the 20% figure in 2016 relates to calves retained for at least 49 days. The recorded increase in the proportion of calves retained in 2019 (24%) relative to 2018 (17%) must also be considered against the corresponding further reduction from 35 to 21 days in the period after which a calf was considered retained. The reduction in the proportion of calves that were subject to confirmatory testing, evident from 2017 onwards (Table 5) contributed to the improvement in these figures and is consistent with a shift to the removal of a greater proportion of animals considered PI based on their initial test values. alongside a focus on testing an increased proportion of animals expected to be TI (reflected in the reduced proportion of those retested that were confirmed as PI). Indeed, of those retained in 2019, 87% had been subject to retest, with this figure being even higher in 2020 (91%), highlighting the benefits of immediate removal based on the initial test result.

The quality of testing is critical to a programme of this nature and using the programme database it was possible to monitor for the occurrence of animals with apparent false negative (AFN) results. The same issue was identified in the Swiss programme, with 57% of identified sources of BVD+ calves born in Phase 3 of that programme being attributed to false negative results (18). A significant proportion of these, beginning in 2016, had a status profile of N-D-P or N-D-P-P, having been detected following the birth of a BVD+ calf to a dam with a prior negative result. The identification of this issue was the basis for changes to the assignment of DAMPI status, requiring all dams of BVD+ calves to be tested independent of their having previously been assigned either an INDINEG or NEG status on the database. This change ensured that this cohort of AFN animals was identified more rapidly than would otherwise have been possible. Measures to identify AFN animals in BVD+ herds were further enhanced from 2019, through the additional testing of all animals on the TASAH sample list generated by the database which had either no known status or only one prior negative (direct or indirect) status recorded.

Despite the identification of these AFN results, the overall quality of the testing remains high. If it is assumed that all AFN animals are genuinely false negative results (as opposed to, for example animals with a TI), the diagnostic sensitivity within the programme, taking into account all steps from sampling to reporting, is 99.45%. While accepting that not all AFN animals will be identified through the database (e.g., some may die without being re-sampled), the overall diagnostic sensitivity remains high, and comparable to that reported elsewhere (18). The specificity of testing is also very high- even if all 805 of the INIPOSINC results recorded in 2020 (Table 2) were considered false positives, this would give a lower limit of specificity of 99.96%.

In contrast to programmes that have followed the Scandinavian model (7), vaccination has not been prohibited in the Irish programme. Genetic diversity of strains in Ireland is limited, with the majority being BVDV-1a (>95% in three studies) followed by BVDV-1b, with single isolates assigned to BVDV-1d and BVDV-1e. There are no reports of BVDV-2 (41–43). Given that the inactivated vaccines predominantly used in the programme both contain BVDV-1a strains, antigenic divergence between field and vaccine strains should therefore not be an issue.

No formal records of vaccine usage are available, but it is accepted to be more frequently applied in dairy herds than in beef herds (44, 45). Analysis of sales data indicated a decline in the number of doses sold over the past 4 years. While these data do not translate directly into the numbers of animals being vaccinated (due to the requirement for two doses of inactivated vaccines for primo-vaccination), it is evident that only a minority of the breeding population are currently vaccinated. This is beneficial from the perspective of the potential to introduce serological screening to the programme at some point as it limits the confounding effects of vaccine-induced antibody at either individual or bulk tank level (46, 47). However, the decline in vaccine usage, in conjunction with the increased naivety of the national herd due to the progress made toward eradication, has raised concerns that while the number of herds with BVD+ births is declining each year the magnitude of these outbreaks may be increasing. The results in Figure 7 show that this is not the case, with the numbers of BVD+ per herd remaining relatively stable throughout the programme. Furthermore, these data highlight that the majority of herds have only one or a small number of BVD+ calves, with both of these findings being considered a reflection of the rapidity with which these animals are identified following the introduction of virus within a tag and test programme.

The eradication programme in Ireland is now entering its final stages. Ireland intends to seek recognition of the programme by the European Commission under the new Animal Health Law (48, 49), with the goal of achieving freedom by 2023. The challenges of completing the final stages of eradication programmes are recognized, particularly with this type of governance structure (36, 50), and a series of further enhancements to the programme have been introduced for 2021 to maintain progress toward this goal. These focus on resolving infection in herds with initial positive results and preventing any further spread from these, while ensuring that the small proportion of herds that have not yet achieved NHS due to the presence of animals of unknown status take the necessary steps to address this. The introduction of legislation in 2020 to require the testing of the small cohort of animals born prior to 2013 into herds which do not yet have NHS is a further contributor to this effort.

As the programme moves toward eradication, the next challenge is to develop mechanisms for post-eradication surveillance, with this now a key focus for the TWG. In contrast to some other EU member states (51), very low numbers of animals are imported into Ireland each year (3,240 in 2019) (52). The fact that the majority of imported animals come from an adjacent country (Northern Ireland) where a tissue-tag based mandatory eradication programme is also in place is anticipated to be an advantage in maintaining a free status post-eradication.

In conclusion, significant progress has been made toward eradication of BVDV in Ireland, with the benefits of this being recognized across multiple sectors (53). This has been achieved through a new governance structure for animal health in Ireland, which has required a sustained collaborative effort between a range of private and public sector stakeholders. The issue of retention of BVD+ calves was the central challenge faced by the programme. A series of incremental changes were made throughout the programme, with these decisions either informed by, or retrospectively supported by the outputs of a series of scientific studies and regular analysis of programme data, highlighting the importance of an objective evidence base for policy decision-making (54).

## Author Contributions

DG and MG prepared the manuscript, which was revised by SM, EL, DB, J-ML, H-HT, PO'S, and SV. EL, DB, J-ML, PO'S, and SV are members of the BVD TWG and/or IG. SM contributed significantly to a number of epidemiological studies which supported the programme. H-HT led the development of the national BVD model. MG is manager for the Irish eradication programme. SV is manager for the Northern Irish eradication programme. All authors contributed to the article and approved the submitted version.

## Conflict of Interest

The authors declare that the research was conducted in the absence of any commercial or financial relationships that could be construed as a potential conflict of interest.

## Footnotes

^1^Available online at: www.icbf.com.

^2^Available online at: https://animalhealthireland.ie/?page_id=229.

^3^Available online at: www.Kynetec.com.

^4^Available online at: www.ecoepi.eu/FarmNet-BVD.
